# Evaluating the Impact of Nutritional Risk on Stress-Induced Hyperglycemia and Trauma Patient Outcomes

**DOI:** 10.3390/healthcare12171746

**Published:** 2024-09-02

**Authors:** Ching-Ya Huang, Yuan-Hao Yen, Ting-Min Hsieh, Ching-Hua Tsai, Shiun-Yuan Hsu, Ching-Hua Hsieh

**Affiliations:** 1 Department of Plastic Surgery, Kaohsiung Chang Gung Memorial Hospital and Chang Gung University College of Medicine, Kaohsiung 83301, Taiwan; b101106030@tmu.edu.tw (C.-Y.H.); bloodguardian@gmail.com (Y.-H.Y.); 2 Department of Trauma Surgery, Kaohsiung Chang Gung Memorial Hospital and Chang Gung University College of Medicine, Kaohsiung 83301, Taiwan; hs168@cgmh.org.tw (T.-M.H.); tsai1737@cloud.cgmh.org.tw (C.-H.T.);

**Keywords:** stress-induced hyperglycemia (SIH), trauma, mortality, Geriatric Nutritional Risk Index (GNRI), nutrition status

## Abstract

Introduction: Stress-induced hyperglycemia (SIH) and malnutrition are common in trauma patients and are linked to worse outcomes. This study examined the influence of nutritional status, determined by the Geriatric Nutritional Risk Index (GNRI), on the incidence of SIH in trauma patients. Methods: A retrospective analysis was conducted on adult trauma patients admitted to a Level I trauma center from 1 January 2009 to December 31, 2021. Patients were categorized into four groups: SIH, diabetic hyperglycemia (DH), diabetic normoglycemia (DN), and non-diabetic normoglycemia (NDN). Nutritional status was assessed using GNRI: high risk (GNRI < 82), moderate risk (82 ≤ GNRI < 92), low risk (92 ≤ GNRI ≤ 98), and no risk (GNRI > 98). Incidence of SIH and outcomes were analyzed across GNRI groups. Results: SIH was associated with higher mortality across all GNRI groups compared to NDN, with the highest rate (45.7%) in the high-risk group. Mortality decreased as GNRI increased in all glucose groups. NDN patients had the lowest mortality rates across GNRI groups. There was no correlation between GNRI and SIH incidence (*p* = 0.259). Conclusion: SIH significantly influenced mortality across all nutritional status groups, with the highest impact in malnourished patients. Although malnutrition did not affect SIH incidence, both SIH and poor nutritional status independently contributed to worse trauma outcomes. Targeted management of hyperglycemia and nutritional deficiencies is crucial for improving survival.

## 1. Introduction

Research indicates that hyperglycemia is a common occurrence, found in 20–30% of trauma patients, with blood glucose levels frequently exceeding 150 mg/dL [1]. In the aftermath of severe trauma, the body emits substantial quantities of stress hormones, which may result in stress-induced hyperglycemia (SIH) [2,3,4,5]. This temporary hyperglycemia, which is defined as blood glucose levels exceeding 200 mg/dL, can occur in patients without a prior history of diabetes as a result of the physiological duress of injury [1,6,7]. The mechanisms consist of insulin resistance, reduced insulin production, and increased glucose production [8]. SIH is independently associated with surgical-site infections in non-diabetic trauma patients [9] and presents as a significant predictor of adverse outcomes in trauma patients [9,10,11,12,13]. It can also worsen the outcomes of trauma patients by enhancing inflammatory responses, increasing the likelihood of bacterial infections, prolonging hospital stays, and causing multiple organ failure [9,10,11,12,13]. SIH is substantially associated with higher mortality rates and worse outcomes among these trauma patients than those with non-diabetic normoglycemia (NDN) or diabetic hyperglycemia (DH) [14,15].

Malnutrition greatly worsens the impact of trauma by compromising immune function, heightening vulnerability to infections, and prolonging the healing process of wounds, resulting in increased illness and death rates in trauma patients [16]. Studies revealed that malnutrition results in poor wound healing and higher infection rates, which compromise immune responses and prolonging inflammation [17,18]. Experimental evidence further shows that malnutrition decreases growth factors essential for wound healing [19]. Li et al. [20] reported that poor nutritional status predicts worse outcomes in severe traumatic brain injury patients. Similarly, Müller et al. [21] found that malnutrition is prevalent in geriatric trauma patients and is linked to worse physical, mental, and cognitive health. With the intervention for nutritional deficiencies to improve trauma recovery outcomes, which is a quick assessment tool for nutritional status, the Geriatric Nutritional Risk Index (GNRI) has been proposed to categorize patients into different risk groups for malnutrition [22,23]. It is calculated using serum albumin levels and the ratio of actual to ideal body weight [24]. The equation for calculating the GNRI is as follows: GNRI = [1.489 × albumin concentration (in grams per liter)] + [41.7 × (weight divided by ideal body weight)]. GNRI has been linked to the prognosis of patients undergoing hemodialysis and those with chronic heart failure [25,26], and it also serves as a predictive indicator for severe ischemic limb amputation [27,28] and is linked to the mortality outcome of trauma patients [22].

Various factors contribute to the occurrence of SIH of trauma patients and impact outcomes. Higher injury severity scores are associated with increased instances of SIH, as the body responds to trauma with elevated glucose production to meet increased metabolic demands [29]. Elevated levels of proinflammatory cytokines and counterregulatory hormones (e.g., cortisol and catecholamines) are key drivers of SIH, contributing to insulin resistance and hyperglycemia [30]. Different trauma types (e.g., blunt vs. penetrating) affect the likelihood and severity of SIH. Blunt trauma tends to evoke a stronger hyperglycemic response than penetrating trauma [31]. However, whether there is an association between malnutrition and the occurrence of SIH was unknown. Therefore, the objective of this study is to examine the influence of nutritional status, as determined by the GNRI, on the incidence and prognosis of SIH in trauma patients. This approach may provide insights into the interplay between nutritional status and glycemic control in trauma care.

## 2. Methods

### 2.1. Methodology and Recruitment of Participants

The protocol for this retrospective investigation was approved by the Chang Gung Memorial Hospital Institutional Review Board (IRB number 202400465B0). Because this study was retrospective in nature, the IRB decided not to require patient permission. Over the course of thirteen years, from 2009 to 2021, we searched through the medical records from the trauma registry data [32]. Adult trauma patients admitted to the hospital through the emergency department and aged 20 and up were included. People who had burns or drowning or hanging injuries or whose trauma registry data were inadequate, were excluded. Specifically, patients with burns, drowning, or hanging injuries were excluded because these types of trauma present unique pathophysiological responses that differ significantly from those caused by other trauma mechanisms. Including these patients could introduce heterogeneity into the study population, potentially confounding the results.

The methodology entailed documenting the medical data of all included trauma patients, including sex, age, presence of other medical conditions, Glasgow coma scale (GCS) scores, injury severity scores (ISS), length of hospital stay, and outcome as discharge or in-hospital mortality. Hyperglycemia was characterized as a blood glucose concentration over 200 mg/dL upon arrival at the emergency department. Diabetes was defined as either having a documented history of diabetes or having a HbA1c level of 6.5% or above during hospitalization according to the American Diabetes Association recommendations or having a verified diagnosis of diabetes through metabolic consultation and subsequent outpatient follow-up. The glucose status of these patients was categorized into four groups based on their glucose levels and the presence of diabetes: SIH (patients without diabetes but with serum glucose levels ≥ 200 mg/dL), DH (patients with diabetes and serum glucose levels ≥ 200 mg/dL), diabetic normoglycemia (DN) (patients with diabetes and serum glucose levels < 200 mg/dL), and NDN (patients without diabetes and serum glucose levels < 200 mg/dL). 

The GNRI was calculated as the formula = [1.489 × albumin (g/L)] + [41.7 × (weight/ideal body weight)], where ideal body weight in this study is calculated as follows: for men, (height in cm − 80) × 0.7, and for women, (height in cm − 70) × 0.6. The trauma patients were also categorized into groups with different nutritional risks according to GNRI, following the recommendations of Bouillanne et al. [24]: High risk of malnutrition (GNRI < 82), moderate risk of malnutrition (GNRI 82 to <92), low risk of malnutrition (GNRI 92 to 98), and no risk of malnutrition (GNRI > 98). The incidence and outcomes of glucose status of the patients (SIH, DH, DN, NDN) in groups of patients with different nutritional status were calculated and analyzed.

### 2.2. Statistical Analysis

The statistical study was conducted using SPSS version 23 of the IBM Windows version. Descriptive statistics were used to compare the features of trauma patients in different GNRI groups and glucose status. Continuous variables were expressed using the mean and standard deviation or the median with interquartile range (IQR), and their comparisons were conducted using one-way analysis of variance (ANOVA). Categorical variables were displayed as frequencies and percentages and examined using chi-square tests. The relationships between groups of glucose status and nutritional risk were analyzed using Pearson chi-square tests. Statistical significance was determined by *p*-values, with a threshold of *p* < 0.05 indicating significant differences.

## 3. Results

### 3.1. Patient Enrollment and Grouping

**Figure 1:**
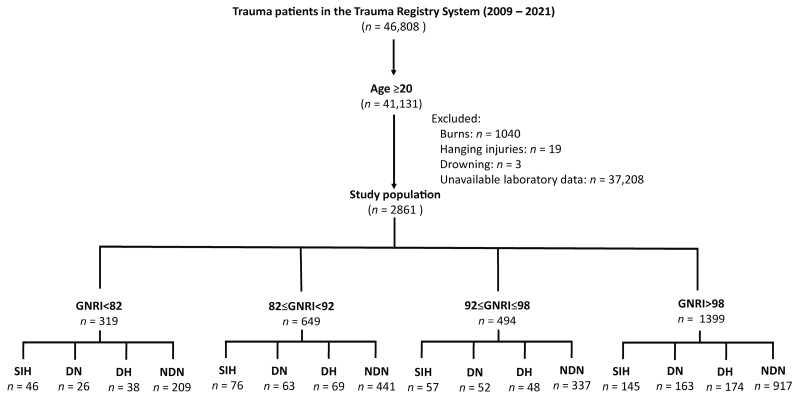
Enrollment and grouping of the patients. Geriatric Nutritional Risk Index (GNRI); SIH = stress-induced hyperglycemia; DN = diabetic normoglycemia; DH = diabetic hyperglycemia; NDN = non-diabetic normoglycemia.

As shown in Figure 1, 41,131 adult patients aged 20 and older were identified from an initial cohort of 46,808 trauma patients. The final study population (*n* = 2861) was determined after the exclusion of burn patients (*n* = 1040), hanging injury patients (*n* = 19), drowning patients (*n* = 3), and those with unavailable laboratory data (*n* = 37,208). The patients were classified into four GNRI categories: GNRI < 82 (*n* = 319) with 46 SIH, 26 DN, 38 DH, and 209 NDN patients; 82 ≤ GNRI < 92 (*n* = 649) with 76 SIH, 63 DN, 69 DH, and 441 NDN patients; 92 ≤ GNRI < 98 (*n* = 494) with 57 SIH, 52 DN, 48 DH, and 337 NDN patients; and GNRI >98 (*n* = 1399) with 145 SIH, 163 DN, 174 DH, and 917 NDN patients. 

### 3.2. Characteristics of Trauma Patients with GNRI < 82 (High Risk of Malnutrition)

Table 1 provides a comparative analysis of trauma patients with GNRI < 82, which is divided according to their glucose status into SIH, DN, DH, and NDN groups. The sex distribution shows no significant differences (*p* = 0.394). SIH patients were notably younger, with a mean age of 58.0 years compared to 66.9 years in NDN patients (*p* = 0.001). Regarding comorbidities, DN patients had a significantly higher prevalence of cerebrovascular accidents (CVA) at 19.2% compared to 5.3% in the NDN group (*p* = 0.004). In DN and DH patients, hypertension (HTN) was significantly more common than in NDN patients. Coronary artery disease (CAD) was also more prevalent in DN patients. SIH patients had significantly lower median GCS scores of 6 (*p* < 0.001), with 58.7% scoring between 3 and 8, compared to the median GCS of 15 in NDN patients. SIH patients also had a higher median ISS of 25 (*p* < 0.001), with 58.7% having severe injuries (ISS ≥ 25) compared to the median ISS of 12 in NDN patients. The hospital stays for SIH patients averaged 25.4 days, though this was not significantly different from the other groups. The SIH and DH groups had a significantly higher mortality rate of 45.7% and 31.6%, respectively, compared to 16.3% in NDN patients.

### 3.3. Characteristics of Trauma Patients with GNRI between 82 and 92 (Moderate Risk of Malnutrition)

As shown in Table 2, DN and DH patients were older, with mean ages of 72.1 and 70.9 years, respectively (*p* < 0.001). Comorbidities were more prevalent in DN and DH patients; HTN was present in 68.3% of DN and 60.9% of DH patients, compared to 28.1% in the NDN group (*p* < 0.001). ESRD was seen in 11.1% of DN and DH patients, compared to 2.9% in the NDN group (*p* < 0.001). GCS scores were lower in the SIH group, with a median of 12, and 46.1% had GCS scores between 3 and 8 compared to the median GCS of 15 in NDN patients (*p* < 0.001). The SIH group had a higher median ISS of 20, with 42.1% experiencing severe injuries (ISS ≥ 25) compared to the NDN group’s median ISS of 16 (*p* < 0.001). In contrast, the DN group had a lower ISS, with a median of 9 compared to the NDN group. SIH patients had longer hospital stays, averaging 26.6 days compared to 20.6 days in the NDN group (*p* = 0.028). Mortality in the SIH group was significantly higher at 23.7% compared to 8.4% in the NDN group (*p* = 0.001).

### 3.4. Characteristics of Trauma Patients with GNRI between 92 and 98 (Low Risk of Malnutrition)

Table 3 focuses on patients with GNRI between 92 and 98. DN and DH patients were older, averaging 72.0 and 68.5 years, respectively, than those patients with NDN. Comorbidities such as HTN were present in 75.0% of DN patients and 64.6% of DH patients, compared to 30.9% in the NDN group (*p* < 0.001). CAD was more prevalent in the DN and DH groups at 15.4% and 18.8%, respectively, compared to 4.5% in the NDN group (*p* < 0.001). SIH patients had significantly lower median GCS scores of 11 (*p* < 0.001) compared to the NDN group’s median GCS of 15. Their median ISS was higher at 20 compared to the NDN group’s median of 16 (*p* = 0.002). The hospital stays were not significantly different among groups. The mortality rate for SIH patients was significantly higher at 22.8% (*p* < 0.001) compared to 3.6% in the NDN group. 

### 3.5. Characteristics of Trauma Patients with GNRI > 98 (No Risk of Malnutrition)

In this group, the DN and DH groups were older than those in NDN. Comorbidities were more prevalent in DN and DH patients, with HTN present in 73.0% of DN and 71.8% of DH patients compared to 29.7% in the NDN group (*p* < 0.001). CAD was seen in 20.2% of DN and 12.1% of DH patients, compared to 4.9% in the NDN group (*p* < 0.001). ESRD was more common in the DN (11.0%) and DH (8.0%) groups compared to 2.6% in the NDN group (*p* < 0.001). SIH patients had a lower median GCS score of 14 (*p* < 0.001) compared to the NDN group’s median GCS of 15. They had a higher median ISS of 22 compared to a median ISS of 13 in the NDN group (*p* < 0.001). SIH patients had longer hospital stays, averaging 22.6 days compared to 15.2 days in the NDN group (*p* < 0.001). The mortality rate for SIH patients was 15.9%, significantly higher than 4.1% in the NDN group (*p* < 0.001). 

### 3.6. The Outcome of Nutritional Status and Its Relationship with Glucose Status

The data from Table 1, Table 2, Table 3 and Table 4 indicate that the mortality rates of NDN patients in the high-risk, moderate-risk, low-risk, and no-risk malnutrition groups were 16.3%, 8.4%, 3.6%, and 4.1%, correspondingly. The mortality rates of the patients with SIH in the high-risk, moderate-risk, low-risk, and no-risk malnutrition groups were 45.7%, 23.7%, 22.8%, and 15.9%, respectively. The findings suggest that nutritional status influences mortality outcomes, with the SIH groups showing a considerably greater mortality rate compared to individuals with NDN, regardless of their nutritional state. In contrast, NDN patients had significantly lower mortality rates, while DN and DH patients had lower mortality rates than SIH but higher than NDN. A crosstabulation of GNRI and glucose status reveals the distribution of patients within each GNRI category across SIH, DN, DH, and NDN groups (Table 5). The Pearson chi-square test found no significant correlation between GNRI categories and glucose status (*p* = 0.259), indicating the distributions were similar across all GNRI levels.

## 4. Discussion

The findings suggest that nutritional status influences mortality outcomes, with the SIH significantly influencing mortality rates across all GNRI groups, with the highest mortality observed in SIH patients. These findings are in accordance with those reports that severe traumatic brain injury patients with SIH had about 50% higher mortality [33]. In the propensity-score-matched patient cohort of patients with traumatic brain injury, Rau et al. [34] highlighted that SIH was linked to a 3.0-fold greater risk of mortality than NDN. These results highlight the severe impact of SIH on trauma prognosis and the need for targeted management strategies to improve survival outcomes. Continuous glucose monitoring and targeted insulin therapy are crucial for managing SIH, yet the optimal glucose target range remains uncertain [35,36]. A personalized, patient-centered approach that balances the risks and benefits for each individual, with close monitoring to avoid both hyperglycemia and hypoglycemia, is recommended [14].

In this study, mortality rates varied notably across different GNRI categories. Patients with GNRI < 82 exhibited the highest mortality rates, with 45.7% for SIH, 23.1% for DN, 31.6% for DH, and 16.3% for NDN. As GNRI scores increased, mortality rates declined, with the lowest mortality rates seen in the GNRI > 98 group. Comparatively, in a study of older trauma patients in intensive care, lower GNRI scores were associated with higher mortality rates, longer hospital stays, and an increased incidence of complications [23]. Patients with a GNRI score below 82 had significantly higher mortality (26.6%) compared to those with higher scores (13.1%), which supports our results [23]. Additionally, research indicates that GNRI can serve as a predictor of complications and outcomes in both elderly and young adult trauma patients, with mortality odds ratios of 6.5 and 2.7, respectively, for elderly and young patients with GNRI < 82, demonstrating its broad applicability in trauma settings [37]. Collectively, the GNRI predicts poor outcomes and underscores the importance of early nutritional assessment and intervention to improve the prognosis of trauma patients [22,38].

Although the study demonstrated that both SIH and GNRI have a significant impact on trauma patients’ outcomes, there appears to be no correlation between the incidence of SIH and GNRI levels. This suggests that the patient’s nutritional status does not directly influence the occurrence of SIH, even though both factors independently affect patient prognosis. As a result, there is a lack of evidence that improving the nutritional status of patients can reduce the occurrence of SIH. Management of SIH and nutritional deficiencies remains critical, with perhaps separate considerations for improving trauma patient outcomes. Integrating the GNRI into trauma care settings offers significant potential to enhance patient outcomes by identifying and managing nutritional risks early in the care process. However, the fast-paced nature of trauma care, with its demand for immediate interventions, presents challenges to the comprehensive application of nutritional assessments. To address this, the GNRI can be employed as a simplified screening tool within the initial trauma evaluation, allowing for quick identification of patients at nutritional risk. These patients can then receive more detailed assessments once their immediate medical needs are stabilized. Resource limitations and the need for specialized training are important considerations, but by equipping existing staff to conduct basic GNRI screenings and implementing standardized protocols, hospitals can efficiently integrate nutritional assessments without disrupting urgent care. Effective application of the GNRI would also require strong coordination between emergency, surgical, and nutritional services to ensure timely and seamless interventions. Despite these challenges, the GNRI’s ability to quickly and accurately assess nutritional risk makes it a valuable tool in improving patient outcomes in trauma settings.

However, it is important to recognize that this study has several limitations. First, it is a retrospective study, which may introduce biases related to data collection and accuracy. Furthermore, only one institution conducted the study, which limited the results’ applicability to different contexts or demographics. Third, we did not account for all potential confounding variables, such as variations in trauma care practices or differences in underlying health conditions among patients. Fourth, the GNRI is based on serum albumin and body weight, which may not fully capture all aspects of nutritional status. Additionally, one of the primary limitations is related to the measurement of blood glucose levels upon the patients’ arrival at the emergency department. Unlike fasting glucose levels or oral glucose tolerance tests, which are the standard for diagnosing hyperglycemia, our approach relied on immediate glucose readings. These measurements may have been influenced by recent dietary or beverage intake, potentially confounding the diagnosis of SIH. This limitation is inherent in the emergency care setting, where immediate and practical assessments are often prioritized over controlled conditions. Notably, there were many formulas used to calculate the ideal body weight. In this study, the ideal body weight is computed in the same way as in prior studies [23], which were based on Taiwanese government suggestions rather than the Lorentz formula from the original publication by Bouillanne et al. [24]. This may cause some bias in the outcome assessment. Lastly, this study did not explore the long-term outcomes of trauma patients, limiting our understanding of the prolonged impact of SIH and malnutrition on recovery and quality of life. Future research should focus on exploring the long-term outcomes of trauma patients who receive early nutritional interventions based on their GNRI scores. Such studies could provide valuable insights into the sustained benefits of addressing nutritional deficiencies early in the care continuum. Additionally, further investigation is needed to explore the impact of specific nutritional supplements or dietary modifications on the incidence and severity of SIH in trauma patients. Conducting multicenter studies would also be beneficial to validate our findings across diverse populations and healthcare settings, thereby refining nutritional intervention protocols and ensuring their broad applicability.

## 5. Conclusions

The study underscores the substantial influence of SIH and nutritional status, as assessed by GNRI, on the outcomes of trauma patients. SIH was associated with an increase in mortality across all GNRI categories, and lower GNRI scores were also associated with higher mortality. Nevertheless, there was no correlation between the incidence of SIH and the levels of GNRI.

## Figures and Tables

**Table 1:** Characteristics of trauma patients with GNRI < 82.

Variables	GNRI < 82 (*n* = 319)				
	SIH *n* = 46	DN *n* = 26	DH *n* = 38	NDN *n* = 209	*p*
Sex					0.394
Male, *n* (%)	26 (56.5)	14 (53.8)	22 (57.9)	138 (66.0)	
Female, *n* (%)	20 (43.5)	12 (46.2)	16 (42.1)	71 (34.0)	
Age, years (SD)	58.0 ± 19.7 *	75.5 ± 9.8	69.9 ± 14.1	66.9 ± 19.9	0.001
Comorbidities					
CVA, *n* (%)	0 (0.0)	5 (19.2) *	1 (2.6)	11 (5.3)	0.004
HTN, *n* (%)	10 (21.7)	16 (61.5) *	26 (68.4) *	70 (33.5)	<0.001
CAD, *n* (%)	2 (4.3)	7 (26.9) *	3 (7.9)	17 (8.1)	0.009
CHF, *n* (%)	1 (2.2)	1 (3.8)	2 (5.3)	5 (2.4)	0.768
ESRD, *n* (%)	3 (6.5)	1 (3.8)	2 (5.3)	9 (4.3)	0.923
GCS, median (IQR)	6 (3–15) *	15 (13–15)	15 (12–15)	15 (12–15)	<0.001
3–8, *n* (%)	27 (58.7) *	2 (7.7)	7 (18.4)	34 (16.3)	<0.001
9–12, *n* (%)	3 (6.5)	4 (15.4)	3 (7.9)	19 (9.1)	0.642
13–15, *n* (%)	16 (34.8) *	20 (76.9)	28 (73.7)	156 (74.6)	<0.001
ISS, median (IQR)	25 (16–29) *	9 (9–16)	16 (9–25)	12 (9–24)	<0.001
1–15, *n* (%)	9 (19.6) *	19 (73.1)	14 (36.8)	114 (54.5)	<0.001
16–24, *n* (%)	10 (21.7)	5 (19.2)	14 (36.8)	45 (21.5)	0.205
≥25, *n* (%)	27 (58.7) *	2 (7.7)	10 (26.3)	50 (23.9)	<0.001
Hospital stays (days)	25.4 ± 21.0	24.5 ± 23.6	26.9 ± 23.8	21.1 ± 17.3	0.223
Mortality, *n* (%)	21 (45.7) *	6 (23.1)	12 (31.6) *	34 (16.3)	<0.001

CVA, cerebrovascular accident; CAD, coronary artery disease; CHF, congestive heart failure; DH, diabetic hyperglycemia; DN, diabetic normoglycemia; ESRD, end-stage renal disease; GCS, Glasgow Coma Scale; HTN, hypertension; IQR, interquartile range; ISS, injury severity score; NDN, non-diabetic normoglycemia; SIH, stress-induced hyperglycemia. * Indicated significant difference in comparison with those in the NDN.

**Table 2:** Characteristics of trauma patients with GNRI between 82 and 92.

Variables	82 ≤ GNRI < 92 (*n* = 649)				
	SIH *n* = 76	DN *n* = 63	DH *n* = 69	NDN *n* = 441	*p*
Sex					0.377
Male, *n* (%)	46 (60.5)	32 (50.8)	39 (56.5)	272 (61.7)	
Female, *n* (%)	30 (39.5)	31 (49.2)	30 (43.5)	169 (38.3)	
Age, years (SD)	57.0 ± 19.1	72.1 ± 12.4 *	70.9 ± 11.5 *	60.9 ± 20.5	<0.001
Comorbidities					
CVA, *n* (%)	3 (3.9)	7 (11.1)	6 (8.7)	17 (3.9)	0.042
HTN, *n* (%)	16 (21.1)	43 (68.3) *	42 (60.9) *	124 (28.1)	<0.001
CAD, *n* (%)	1 (1.3)	9 (14.3)	8 (11.6)	28 (6.3)	0.010
CHF, *n* (%)	0 (0.0)	1 (1.6)	4 (5.8) *	5 (1.1)	0.019
ESRD, *n* (%)	1 (1.3)	7 (11.1) *	8 (11.6) *	13 (2.9)	<0.001
GCS, median (IQR)	12 (5–15) *	15 (13–15)	15 (12–15)	15 (10–15)	<0.001
3–8, *n* (%)	35 (46.1) *	4 (6.3)	11 (15.9)	85 (19.3)	<0.001
9–12, *n* (%)	4 (5.3)	8 (12.7)	7 (10.1)	64 (14.5)	0.141
13–15, *n* (%)	37 (48.7) *	51 (81.0)	51 (73.9)	292 (66.2)	<0.001
ISS, median (IQR)	20 (13–26) *	9 (9–16) *	9 (9–21)	16 (9–22)	<0.001
1–15, *n* (%)	22 (28.9) *	42 (66.7) *	38 (55.1)	206 (46.7)	<0.001
16–24, *n* (%)	22 (28.9)	15 (23.8)	16 (23.2)	143 (32.4)	0.270
≥25, *n* (%)	32 (42.1) *	6 (9.5)	15 (21.7)	92 (20.9)	<0.001
Hospital stays (days)	26.6 ± 24.8 *	24.0 ± 23.0	22.8 ± 16.9	20.6 ± 14.8	0.028
Mortality, *n* (%)	18 (23.7) *	10 (15.9)	9 (13.0)	37 (8.4)	0.001

CVA, cerebrovascular accident; CAD, coronary artery disease; CHF, congestive heart failure; DH, diabetic hyperglycemia; DN, diabetic normoglycemia; ESRD, end-stage renal disease; GCS, Glasgow Coma Scale; HTN, hypertension; IQR, interquartile range; ISS, injury severity score; NDN, non-diabetic normoglycemia; SIH, stress-induced hyperglycemia. * Indicated significant difference in comparison with those in the NDN.

**Table 3:** Characteristics of trauma patients with GNRI between 92 and 98.

Variables	92 ≤ GNRI ≤ 98 (*n* = 494)				
	SIH *n* = 57	DN *n* = 52	DH *n* = 48	NDN *n* = 337	*p*
Sex					0.009
Male, *n* (%)	35 (61.4)	31 (59.6)	19 (39.6) *	219 (65.0)	
Female, *n* (%)	22 (38.6)	21 (40.4)	29 (60.4) *	118 (35.0)	
Age, years (SD)	56.9 ± 19.6	72.0 ± 11.8 *	68.5 ± 13.6 *	58.6 ± 20.2	<0.001
Comorbidities					
CVA, *n* (%)	1 (1.8)	14 (26.9) *	6 (12.5)	18 (5.3)	<0.001
HTN, *n* (%)	17 (29.8)	39 (75.0) *	31 (64.6) *	104 (30.9)	<0.001
CAD, *n* (%)	4 (7.0)	8 (15.4) *	9 (18.8) *	15 (4.5)	<0.001
CHF, *n* (%)	0 (0.0)	0 (0.0)	2 (4.2)	8 (2.4)	0.312
ESRD, *n* (%)	0 (0.0)	4 (7.7)	3 (6.2)	14 (4.2)	0.210
GCS, median (IQR)	11 (4–15) *	15 (13–15)	15 (10–15)	15 (11–15)	<0.001
3–8, *n* (%)	24 (42.1) *	7 (13.5)	7 (14.6)	60 (17.8)	<0.001
9–12, *n* (%)	8 (14.0)	4 (7.7)	7 (14.6)	30 (8.9)	0.403
13–15, *n* (%)	25 (43.9) *	41 (78.8)	34 (70.8)	247 (73.3)	<0.001
ISS, median (IQR)	20 (13–29) *	9 (9–24)	13 (9–19)	16 (9–22)	0.002
1–15, *n* (%)	16 (28.1) *	29 (55.8)	28 (58.3)	159 (47.2)	0.006
16–24, *n* (%)	17 (29.8)	10 (19.2)	10 (20.8)	109 (32.3)	0.128
≥25, *n* (%)	24 (42.1) *	13 (25.0)	10 (20.8)	69 (20.5)	0.005
Hospital stays (days)	23.3 ± 18.8	19.4 ± 15.2	23.5 ± 15.7	19.2 ± 14.9	0.116
Mortality, *n* (%)	13 (22.8) *	5 (9.6)	4 (8.3)	12 (3.6)	<0.001

CVA, cerebrovascular accident; CAD, coronary artery disease; CHF, congestive heart failure; DH, diabetic hyperglycemia; DN, diabetic normoglycemia; ESRD, end-stage renal disease; GCS, Glasgow Coma Scale; HTN, hypertension; IQR, interquartile range; ISS, injury severity score; NDN, non-diabetic normoglycemia; SIH, stress-induced hyperglycemia. * Indicated significant difference in comparison with those in the NDN.

**Table 4:** Characteristics of trauma patients with GNRI >98.

Variables	GNRI > 98 (*n* = 1399)				
	SIH *n* = 145	DN *n* = 163	DH *n* = 174	NDN *n* = 917	*p*
Sex					0.001
Male, *n* (%)	103 (71.0)	89 (54.6)	90 (51.7) *	575 (62.7)	
Female, *n* (%)	42 (29.0)	74 (45.4)	84 (48.3) *	342 (37.3)	
Age, years (SD)	52.7 ± 17.6	68.8 ± 10.8 *	67.0 ± 12.1 *	53.7 ± 19.0	<0.001
Comorbidities					
CVA, *n* (%)	7 (4.8)	20 (12.3) *	14 (8.0)	38 (4.1)	<0.001
HTN, *n* (%)	43 (29.7)	119 (73.0) *	125 (71.8) *	272 (29.7)	<0.001
CAD, *n* (%)	5 (3.4)	33 (20.2) *	21 (12.1) *	45 (4.9)	<0.001
CHF, *n* (%)	1 (0.7)	4 (2.5) *	5 (2.9) *	3 (0.3)	0.002
ESRD, *n* (%)	1 (0.7)	18 (11.0) *	14 (8.0) *	24 (2.6)	<0.001
GCS, median (IQR)	14 (5–15) *	15 (15–15)	15 (13–15)	15 (13–15)	<0.001
3–8, *n* (%)	55 (37.9) *	11 (6.7) *	20 (11.5)	134 (14.6)	<0.001
9–12, *n* (%)	12 (8.3)	15 (9.2)	18 (10.3)	79 (8.6)	0.890
13–15, *n* (%)	78 (53.8) *	137 (84.0)	136 (78.2)	704 (76.8)	<0.001
ISS, median (IQR)	22 (9–27) *	9 (9–16)	15 (9–20)	13 (9–20)	<0.001
1–15, *n* (%)	46 (31.7) *	100 (61.3)	87 (50.0)	506 (55.2)	<0.001
16–24, *n* (%)	38 (26.2)	46 (28.2)	54 (31.0)	247 (26.9)	0.706
≥25, *n* (%)	61 (42.1) *	17 (10.4)	33 (19.0)	164 (17.9)	<0.001
Hospital stays (days)	22.6 ± 24.6 *	17.0 ± 15.5	18.6 ± 15.6 *	15.2 ± 13.8	<0.001
Mortality, *n* (%)	23 (15.9) *	13 (8.0)	11 (6.3)	38 (4.1)	<0.001

CVA, cerebrovascular accident; CAD, coronary artery disease; CHF, congestive heart failure; DH, diabetic hyperglycemia; DN, diabetic normoglycemia; ESRD, end-stage renal disease; GCS, Glasgow Coma Scale; HTN, hypertension; IQR, interquartile range; ISS, injury severity score; NDN, non-diabetic normoglycemia; SIH, stress-induced hyperglycemia. * Indicated significant difference in comparison with those in the NDN.

**Table 5:** Crosstabulation of glucose status and Geriatric Nutritional Risk Index (GNRI).

			Glucose Status				Total
			SIH	DN	DH	NDN	
GNRI	<82	*n*	46	26	38	209	319
		%	14.4%	8.2%	11.9%	65.5%	100.0%
	82 to <92	*n*	76	63	69	441	649
		%	11.7%	9.7%	10.6%	68.0%	100.0%
	92–98	*n*	57	52	48	337	494
		%	11.5%	10.5%	9.7%	68.2%	100.0%
	>98	*n*	145	163	174	917	1399
		%	10.4%	11.7%	12.4%	65.5%	100.0%
Total		*n*	324	304	329	1904	2861
		%	11.3%	10.6%	11.5%	66.6%	100.0%
*p*-value (two-sided) of chi-square test is 0.259							

## Data Availability

The de-identification data could be provided upon request for academic research purposes.
